# Qualitative Evaluation of a Novel Educational Tool to Communicate Individualized Hip Fracture Prognostic Information to Patients and Surrogates: My Hip Fracture (My-HF)

**DOI:** 10.1177/21514593211050513

**Published:** 2021-10-23

**Authors:** Corita Vincent, Pete Wegier, Vincent Chien, Allison Miyoshi Kurahashi, Shiphra Ginsburg, Hedieh Molla Ghanbari, Jesse Isaac Wolfstadt, Peter Cram

**Affiliations:** 1 Department of Medicine, 12366 University of Toronto, Toronto, ON, Canada; 2 Temmy Latner Centre for Palliative Care, 518775 Sinai Health System, Toronto, ON, Canada; 3 Division of General Internal Medicine and Geriatrics, 518775 Sinai Health System, Toronto, ON, Canada; 4 Department of Family and Community Medicine, 12366 University of Toronto, Toronto, ON, Canada; 5 Granovsky Gluskin Division of Orthopaedic Surgery, 518775 Sinai Health System, Toronto, ON, Canada; 6 Department of Surgery, 7938 University of Toronto, Toronto, ON, Canada; 7 Institute for Health Policy, Management and Evaluation, University of Toronto, Toronto, ON, Canada; 8 Division of General Internal Medicine and Geriatrics, University Health Network, Toronto, ON, Canada

**Keywords:** risk communication, health education, patient education tool, hip fracture

## Abstract

**Introduction:**

Mortality and morbidity are high for older adults after hip fracture (HF), but patients and surrogate decision makers (SDMs) are typically unaware of the poor prognosis. We developed a novel educational tool, *My Hip Fracture* (My-HF), to provide patients and SDMs of patients hospitalized with acute HF individualized estimates of their post-HF prognosis. We conducted initial usability testing of My-HF in a sample of patients with HF and SDMs.

**Materials and Methods:**

My-HF provides information about: 1) anatomy and risk factors for HF; 2) Hip fracture treatment received; 3) individualized predicted risk of adverse events and 4) anticipated discharge trajectory. We conducted a qualitative usability study using a convenience sample of hospitalized, post-operative patients with acute HF or SDMs of patients who lacked decision-making capacity. We used semi-structured interviews to obtain feedback. Thematic analysis was used to identify themes and concepts.

**Results:**

We conducted interviews with 8 patients and 9 SDMs (mean age of interviewees 70.1 years, 41% female). My-HF was generally well received. Thematic analysis identified legibility and visual appeal, comprehension, numeracy, utility and reflection as prominent themes. Most respondents found My-HF to be useful in improving their understanding of HF and as a potential mechanism for sharing information with other care team members (including family and professionals). Suggestions for improvement of legibility, presentation of the individualized prognosis information and content were identified.

**Discussion:**

Patients and SDMs are generally accepting of My-HF and found it useful for communicating individualized prognostic information. Feedback identified areas for improvement for future iterations of the tool.

**Conclusion:**

My-HF presents a means of addressing the gap in understanding of prognosis post-HF as a part of patient-centered care. Further evaluation will be needed to assess the impact of My-HF on patient and SDM reported outcomes as we transition from a paper to smart-phone enabled web application.

## Introduction

Low impact hip fracture (HF) remains a major cause of morbidity and mortality for older adults worldwide, despite improvements in care over the past 20 years.^1-4^ Not only are patients with HF subject to a 3- to 8-fold increase in mortality in the first 30 days, but survivors also have increased mortality rates up to 10 years post-fracture compared to peers who did not experience a fracture.^5,6^ Further, frail elders who survive fracture typically have persistently reduced function even after intensive rehabilitation.^7-9^

Prior research suggests that patients and surrogate decision makers (SDMs) have limited understanding of the seriousness of HF, including limited awareness of risk of death and limited functional recovery.^10-13^ This discrepancy is concerning because it violates foundational principles of patient-centered care.^14,15^ Additionally, lack of understanding of illness prognosis and severity can be associated with frustration, anxiety and decision regret.^16,17^ There is a growing body of literature suggesting the need for earlier introduction of palliative care and advanced care planning (ACP) for frail elders with an array of conditions, including HF.^18,19^ Lack of understanding of HF prognosis has significant implications for patient and SDM readiness to engage in ACP.^20^

Recognizing a gap in current HF care, we assembled a multi-disciplinary team to develop a novel educational tool (My Hip Fracture [My-HF]) to improve communication about HF treatment and convey individualized prognostic information to patients and SDMs of patients hospitalized with acute HF. We describe the development of My-HF and the results of qualitative usability testing conducted in a sample of patients with HF and their SDMs.

## Materials and Methods

### My-HF Description

We convened a multi-disciplinary team of geriatricians, orthopaedic surgeons, hospitalists, physiotherapists, palliative care specialists and medical decision scientists to develop My-HF over a 3-year period. First, we conducted a foundational study to explore patient and SDM understanding of HF along with gaps in knowledge.^10^ Based upon our findings and input from our team, an initial paper-based draft of My-HF was built, recognizing that the paper instrument would ultimately be converted to a smart-phone enabled web-app for eventual widespread evaluation. Key information was presented in 4 discrete sections: 1) hip anatomy and HF risk factors; 2) primary HF treatment; 3) individualized predicted risk of major adverse event within 30 days and discharge to post-acute care and 4) anticipated post-HF discharge trajectory. Drafts were circulated amongst team members, reviewed and refined iteratively though in-person meetings and email until a suitable draft was developed [see Supplemental File 1].

The individualized predicted risk section [see Supplemental File 1, Section 3] was designed to facilitate clinicians’ communication of complication rates for individual patients, easily calculated based upon demographic and clinical risk factors using the American College of Surgeons National Surgical Quality Improvement Program (ACS-NSQIP) risk calculator^21-23^ The risk calculator is publicly available and commonly used in the surgical setting, but the output is not designed for patients. We designed My-HF to facilitate communication of individualized risk of 2 key adverse outcomes in a user-friendly, patient-centered fashion: 1) death or major complication within 30 days of surgery and 2) discharge to post-acute care (rehabilitation or long-term care). Major complication included cardiac arrest, myocardial infarction, pneumonia, renal failure/progressive renal insufficiency, pulmonary embolism, deep vein thrombosis, return to operating room, surgical site infection, sepsis, unplanned intubation and wound disruption-all as defined by the ACS-NSQIP. We considered several numerical and graphical options for presenting risk, ultimately choosing percentage risk represented numerically and on a thermometer bar. This technique incorporates both numerical estimate and visual aid, both of which enhance comprehension.^24,25^ Because the goal of this study was to assess the usability and acceptability of My-HF, patients and SDMs were not provided with their individualized risk estimates. Our tool was designed for a grade 6 reading level.

### Study Population

We assessed the usability of our paper-based instrument through semi-structured interviews with a convenience sample of patients aged ≥65 years, hospitalized with acute isolated HF on the orthopaedic service in a major Toronto teaching hospital between May and December 2019. Patients were excluded if: 1) unable to provide informed consent and their SDM was unavailable or 2) unable to communicate in English and lacked an English-speaking SDM. For patients who had cognitive impairment (identified as a Mini-Cog score of <3^26^ or documented severe dementia), psychiatric illness, or a significant language barrier, we sought SDM participation reflecting that in clinical practice My-HF would be administered to SDMs when patients lack capacity. This study was approved by the research ethics board (IRB), and all methods were performed in accordance these guidelines and with the Declaration of Helsinki. Informed written consent was obtained from all participants prior to study participation.

### Data Collection

Patients and SDMs participated in semi-structured, audio recorded, in-person interviews conducted by research team members (AK or CV) to explore reaction to My-HF using a structured interview guide [see Supplemental File 2]. We collected basic participant demographics: age, sex, educational attainment and relationship to patient (for SDMs). Field notes were reviewed and discussed, and the interview guide was iteratively revised to explore emerging themes. Data collection continued until no new themes or concepts arose and thematic saturation was achieved.^27^ Interviews were conducted in hospital setting, prior to patient discharge; duration was 15–45 minutes.

### Data Analysis

We used a constructivist framework to guide analysis.^28,29^ Interview recordings were transcribed verbatim and analyzed using MaxQDA qualitative software (VERBI Software, Berlin, Germany). Inductive thematic analysis was used to identify themes and concepts. All investigators participated in developing an initial coding scheme and identify themes, and 2 team members (AK and CV) subsequently refined code definitions and hierarchies by coding 4 representative interviews. Differences in coding were reconciled by consensus. The remaining interviews were coded by CV. Coding and emerging themes were discussed and updated at regular team meetings. Finally, we re-grouped transcripts by participant type (patient vs SDM) and re-read them to see if themes varied by role.^28^

## Results

**Figure 1.**
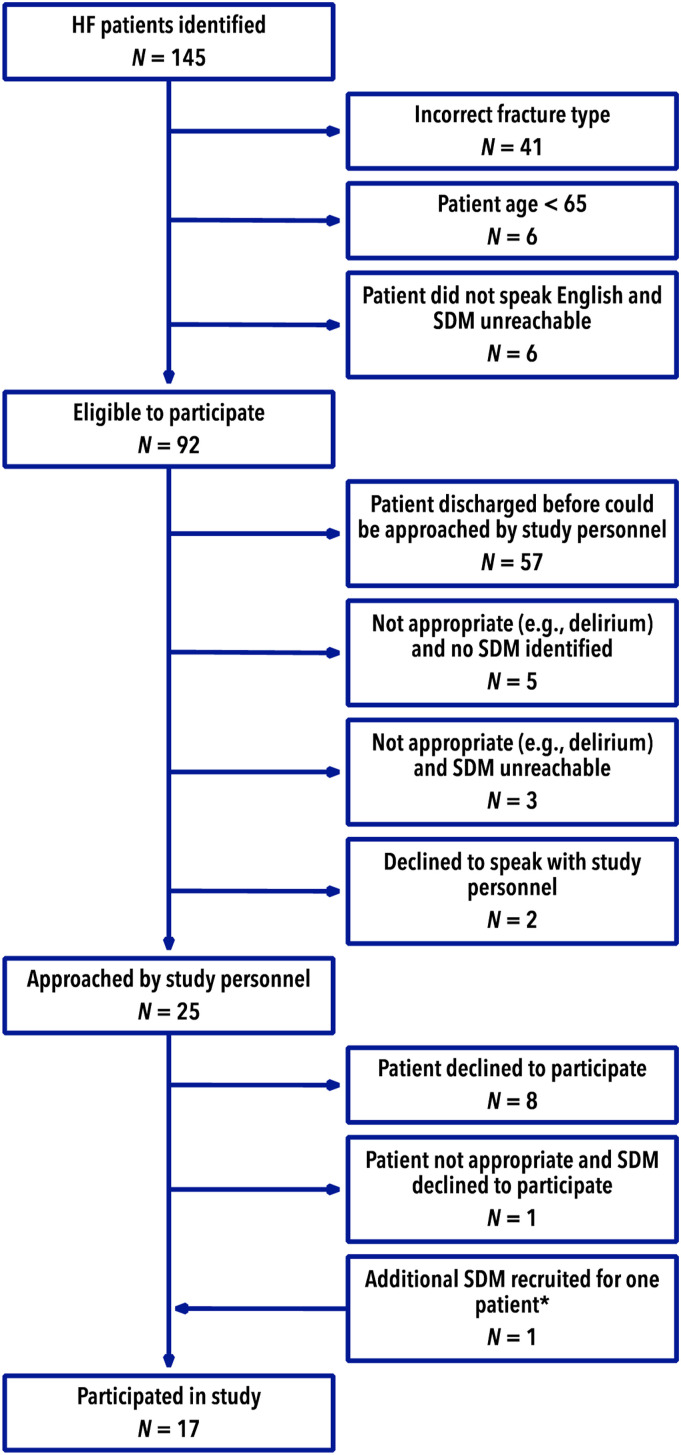
Participant identification and exclusion. HF-Hip fracture, SDM-surrogate decision maker.

We interviewed 8 patients with HF (mean age 76 years, 62.5% women) and 9 SDMs (mean age 63.5 years, 33.3% women) (Figure 1 and Table 1).

**Table 1.** Participant Demographics.

*Participant Characteristics*		
	Patients	SDMs
*N*	8	9*
Interviews	8	8
Age—Mean (SD)**	76 (9)	64 (14)
Female—*N* (%)	5 (63)	3 (33)
Education**—*N* (%)		
Less than high school	1 (13)	0 (0)
High school	3 (38)	1 (11)
Some university/college	1 (13)	1 (11)
University/college degree	2 (25)	3 (33)
Graduate or professional degree	1 (13)	3 (33)
Relationship to patient—*N* (%)		
Spouse	NA	3 (33)
Child	NA	5 (56)
Grandchild	NA	1 (11)

SD-standard deviation, SDM-surrogate decision maker.

NA-not applicable.

*2 SDMs participated jointly in a single interview.

**Missing data: SDM data for age for 2 participants and education of 1 participant.

We identified 5 themes with related subthemes described below with exemplar quotes. The source for each quote (patients [P], SDM [SDM], interviewer [I]) is shown.

### Theme 1: Legibility and Visual Appeal

Most participants felt that the lay-out, colours and images were clear and legible; however, some issues with font size and coloured backgrounds were identified. Patients, particularly those who did not have their corrective lenses, were more likely than SDMs to identify concerns.

‘*I think that the font size is nice for me.’ (SDM 05)*

*‘P: Well, I can’t read it without glasses. I: Okay. Can you read this [larger font]? P: Oh yeah’. (P 15)*

*Suggestion for improvement:* Simplify the colour scheme.

‘*Anything for people with [eye-related diseases], anything white and black is the best’ (P 06)*

### Theme 2: Comprehension

#### Terminology

Most participants found the tool easy to understand and that explanations of medical terminology improved clarity.

‘*Well, it’s very clear, and I can read the different parts of the anatomy. And the risk factors are very easy’. (P 01)*

Certain medical terms (hemiarthroplasty, arthroplasty, comfort care, long-term care, acute care, rehabilitation and palliative care) were cited as confusing by multiple subjects.

‘*Comfort care. I don’t really know what they mean by it. What exactly does it mean’? (P 09)*

*Suggestions for improvement:* Replace unclear terminology or provide explanations.

‘*So you might want to say ‘hospital’ as opposed to ‘acute care’’. (SDM 08)*

‘*You could have that word, but underneath it, a description what it is’ (SDM 02)*

#### Images

Respondents felt most images improved understanding, but 2 were identified as unclear (Table 2 and Supplemental File 1).

‘If you don’t know what they’re talking about-- when you see it on the [picture] maybe it can give you some understanding’. (SDM 07)

**Table 2.** Graphics That Were Felt to Impair Understanding.

Graphic	Intended to convey	Exemplar quotation
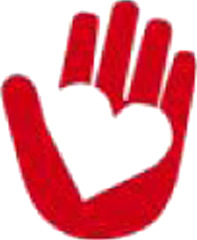	Comfort care	‘Well, the picture looks as though you’re supposed to [love] your hand, but I didn’t have an operation on my hand’. (P 09)
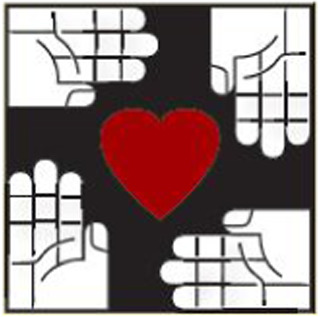	Risk of adverse event within 30 days	‘But I’m not really sure what this picture is supposed to [tell me]’ (P 01)

P – Patient.

*Suggestions for improvement:* Participants requested alternative images for those they found unclear.

‘*[Pointing to 4 hands and heart graphic] This one here, I don’t know, maybe you can change the picture’. (SDM 02)*

### Theme 3: Numerical Understanding

Most participants had a general understanding that in Section 3 (Supplemental File 1), a higher percentage implied greater risk of an adverse outcome. A few participants had a very good understanding of risk as presented.

‘*Okay. So if it’s 95%, only 1 out of 20 cases will not experience a complication. So very likely, very high risk’. (SDM 05)*

However, many participants made at least 1 error in interpretation (Table 3); errors were commonly identified by the interviewer and infrequently self-identified by the participant.

**Table 3.** Errors in Interpretation of Quantitative Risk of Adverse Outcomes.

Error	Exemplar Quotation
Applying percentage risk to a different outcome	‘I: what if it was at 95% [risk of rehab or long-term care]? P: ‘I would probably want to talk to someone just to find out how long they think or why it would need that long’’ (P 03)
Applying percentage risk of adverse event to each adverse event equally	‘Maybe you could have an 85% chance of pneumonia, but only a 50% of bedsores, and a 35% chance of delirium. I don’t see how one percentage could apply to all these different risk factors’. (P 14)
Assuming smaller percentage implied greater risk	‘I: What would that mean to you if I said 15%? P: ‘I’d be sad… if you told me 15%, I would assume it was on the negative.’’ (P 09)
Unable to apply information	‘How serious is 30%? Is everybody 30%? Is that a normal thing? … I’d want to know how you got to the 30% and whether I should be worried about the 30%’ (SDM 08)

I-interviewer, P-Patient, SDM-surrogate decision maker.

*Suggestions for Improvement:* Discussion revealed that addition of an anchor (representing ‘average’ or ‘baseline’ risk) would be helpful for contextualizing individual risk.

‘*I: So would it be helpful to [have] a bar that says, ‘The average risk is here and your risk is here or here?’ SDM: If you can do that, it would be really helpful. Yeah. I think that would be quite good actually’. (SDM 08)*

### Theme 4: Utility

While most participants found My-HF useful, some found it to be of more limited value (Table 4). Most participants felt that the tool was useful in providing an opportunity to review or consolidate knowledge and as a mechanism for sharing information about their HF with friends, family, and other health care providers. Some thought the information would facilitate conversations with family or their care team about ACP or motivate behaviour change. Some felt the tool, or aspects of the tool, were less useful because: 1) information interpreted as not useful or confusing; 2) information was already known; or 3) information was discordant with what respondents desired.

**Table 4.** Perceived Utility of the My-HF tool.

Exemplar Quotation	
**Positive utility**	
Chance to learn or review information	‘It’s better that everybody’s given a pamphlet like this so they could see what the injured-- which part, and have that understanding. It’s educating people more, I think’. (SDM 02)
Reference point	‘We’d use this to… actually sit down with other members of the family or friends or whatever you need be and say, "Here’s where we’re at.” And you’re not going to miss the point or you’re not going to give information that’s not correct’. (SDM 10)
Prompt conversation	‘And the next step in my mind would be someone from the care team…will sit down with you and go through all of this’. (SDM 08)
Change behaviour	‘So I suppose seeing that and knowing that those were factors tells me that I have to be more careful’. (P 14)
**Negative utility**	
**Content related**	
I already know this	‘Yes. Yes. They have been very well informed and told me that I’m doing well. And I think they do everything that’s possible’. (P 06)
Content is of uncertain practical use	‘It is what it is. And probably those have been the facts for a long time. And now that I’m hearing it, is it going to change an outcome? I don’t think so’. (SDM 05)
Mismatch between information provided and desired	‘I see here sort of four different ways of holding the joint together once it’s broken. And I don’t really care. All we really care about is functionality. I mean, that’s sort of the main thing. Can she walk again’? (SDM 13)
**Presentation related**	
Information is poorly understood and therefore not useful	‘So I think it’s very unclear. I don’t think it’s-- I don’t think it’s something I’d-- I just don’t see the point of giving that to somebody’. (P 14)
Dislike single percentage apply to multiple items	‘I mean, obviously, the risk of death is of more of concern than the risk of a urinary tract infection which can be treated, right’? (P 11)
Dislike percentage referring to discharge destination	‘So I’m just not sure that that gives me, as a percentage, any useful information. If anything, it makes me think ‘Well, Christ, you should know yes or no.’ What kind of care do I need’? (SDM 08)

P-Patient, SDM-surrogate decision maker.

*Suggestions for improvement:* Split composite endpoint into the probabilities for individual components (e.g., death separated from non-death outcomes).

‘*75%, I don’t know if it’s death or the-- so I would definitely need which one-- like, for me, each picture needs specific numbers, each one of them’. (SDM 07)*

Remove section 3B ‘need for rehab or LTC after acute care’ altogether as most patients know their discharge destination by this time and applying a probability was perceived as confusing.

‘*If you’ve already decided what I need, then why do I need my probability? … I might think about dropping that [section 3B] and just moving on to this is what—based on our assessment, this is the best course of action for you to follow’. (SDM 08)*

A deeper discussion with their health care team about the My-HF individualized prediction information, particularly if the prediction was worrisome.

‘*No. I’d want somebody to explain it to me if it was that high. If it was like 50%, then written information would be fine. But anything above that, I think you need someone to explain it to you’. (P 03)*

Some participants suggested that My-HF results would best be reviewed prior to surgery, others after or that timing was less important.

‘*You could get it before or after. Before, you’re really not going to know too much. But after, no, I think it would be very helpful either way’. (P 03)*

### Theme 5: Reflection

#### Affect

Some participants felt that presentation of individualized prognosis data would produce negative emotions—such as sadness, worry, or fear—in themselves and/or others. Others had a positive response, expressing appreciation for inclusion of individualized risk and these sensitive topics.

‘*To me, that’s a little bit not depressing, but-- I don’t know. Some people take it differently. I don’t know. I wouldn’t like that’. (P 02)*

‘*I mean, if you’re telling me ‘Okay, it’s time to move your spouse into palliative care—' yeah, I mean it’s a fact of life. I don’t think there’s any issues with that at all’. (SDM 08)*

#### Applicability to Self

Participants frequently reflected on how and if the information presented related to their current situation. Participants who were more accepting commonly noted that My-HF was useful precisely because ‘it applies to me’, while respondents who felt the information did not apply to them more commonly reported inutility.

‘*I: Do you find that information helpful or useful? P: Yeah, it applies to me” (P 09)*

*“The surgical complications. No, I don’t think I’ll have any urinary tract infections. Blood clots, they have taken care of. And bedsores, I don’t have any. And no deliriums [laughter]. And pneumonia, I have had the flu shot’. (P 06)*

#### Past Experiences

Participants commonly contextualized the My-HF content in the form of narratives, of either their own past medical experience, or of other people in their lives who had experienced HF or something similar.

‘*This is good education because my father-in-law went through the same thing. He got bedsores. We didn’t know he was going to get bedsores. He also got urinary infection, and then he was delirious. And not only that, he died’. (SDM 02)*

#### Expression of Future Goals

Upon review of My-HF some participants spontaneously verbalized thoughts about goals for the future, including goals of care.

‘*it’s very good because people need to know what they’re up against. And someone might say, ‘Forget it, and don’t bother’’. (P 01)*

#### Evidence of Knowledge Gap—Surprise at Content of the Tool

Participants also frequently reflected on information imparted by My-HF that they found surprising or did not previously realize.

‘*I’d just never thought of things like that. And I’d never think I would have to. Well, you just go home like a broken ankle or a broken [laughter] wrist or something. But you don’t think when it’s your hip or something, so no, [the tool] is good’. (P 03)*

## Discussion

We assessed the usability and acceptability of My-HF in a sample of patients and SDMs of patients hospitalized with acute HF. Respondents reported My-HF to be useful in improving their understanding of HF and as a mechanism for sharing information with family and their health care team. Participants also viewed the inclusion of individualized risk estimates as valuable in starting conversations about prognosis and prompting reflection about goals of care.

My-HF should be considered in the context of current care of older adults with low impact HF. Many recent efforts to improve HF care have focused on the medical and surgical aspects of treatment. Efforts have included initiatives to reduce the delay in time to surgical repair, defining the optimal type of repair (e.g., hemiarthroplasty vs total hip arthroplasty) and choice of anesthesia (regional vs general), and incorporating and evaluating surgical co-management between orthopaedic surgeons, geriatricians, and hospitalists.^30-33^ There has been far less attention to addressing the gap in patient and caregiver understanding of HF prognosis.^10,11^ While the internet provides many publicly available resources that discuss HF treatment and recovery,^34,35^ these resources are not tailored to the prognosis or risk of the individual patient. Alternatively, the ACS-NSQIP risk calculator provides individualized information regarding prognosis but is not designed for patients or SDMs with limited medical knowledge or numeracy.^36^ The importance of both individualized information and tailoring to numeracy and literacy is well-established in the patient education literature.^37-39^

The lack of tailored HF educational materials belies a general lack of attention to patient-centred care for a condition that is often misconstrued as solely mechanical and fixed with surgery. The acute care of HF frequently overlooks the increased mortality risk that persists in seniors month-to-years after the inciting fracture is repaired. Likewise, despite a growing movement to view HF as an opportunity to discuss prognosis and ACP, communication of the increased risk of adverse outcomes has received limited attention.^40,41^ Patient-centred HF care should involve a partnership between the care team and patients and SDMs that begins with conveying treatment and prognosis information in ways that they can understand.^14^ Patient education is foundational to patients (and SDMs) having the information needed to actively engage in their care, including acute illness management and ACP. For those who are not ready to engage in care decisions, including ACP, patient education (or consciousness raising) interventions are important in early stages of behaviour change to progress patients and SDMs towards engagement.^42,43^ Additionally, evidence from other diseases and conditions suggests that patient education about illness prognosis is associated with decreased anxiety, regret and even improvement in overall quality of life measures.^16,17^ Thus, the development of My-HF represents a novel effort to integrate a well-studied risk prediction model (ACS-NSQIP) into a state-of-the art HF educational tool to bridge the gap between the clinical care team’s and the patient’s (or SDM’s) understanding of HF trajectory.

It is important to comment on the specific feedback we received from patients and SDMs in our testing. Despite careful attention to literacy and numeracy during initial development of My-HF, patients and SDMs cited difficulty with medical jargon, unfamiliar terms (e.g., comfort care), and with certain graphics. This feedback reinforces the need for usability testing and will guide changes in the next iteration of our tool. Many patients and SDMs had difficulty understanding aspects of the individualized prognostic information including the interpretation of the composite endpoint and how the numerical risk applied to their personal circumstance. Difficulty with probabilistic risk interpretation is consistent with prior research, even among highly educated samples.^44,45^ In reviewing My-HF, participants requested a reference to help in their understanding, which has been previously shown to improve patient understanding of numerical data.^46^ Adding anchors or interactive simulated experiences to future versions of My-HF should help enhance understanding of risk prediction. While the provision of individualized risk estimates was viewed favorably, some participants were surprised by the range of possible adverse outcomes, especially death. Our finding that patients and SDMs are accepting of individualized HF prognostic information, even if somewhat alarmed, is consistent with studies from other conditions.^47,48^

### Limitations

First, our study was conducted in a single academic medical center and should be generalized with care. Second, health literacy and numeracy of participants were not formally evaluated, but this mimics how My-HF would be used in actual real-world clinical practice. Finally, our study asked participants to review My-HF as a hypothetical clinical tool and did not provide them with their personal individualized prognostic information, consistent with the goals of our usability testing. Future studies are required to evaluate the impact of My-HF when providing patients and SDMs with their actual prognostic information and assessing the impact of My-HF on outcomes including understanding, satisfaction and readiness to engage in advanced care planning.

### Next Steps

We are revising My-HF based upon the feedback we received. Several of the issues identified by participants were inherent to our paper-based tool and will be easily addressed as we convert our paper instrument to a smart-phone enabled web application. For example, customizable settings will allow users to see only the treatment and discharge information applicable to them and adjust font size and background for the visually impaired. Medical jargon will be removed or clarified. Specifically, ‘Palliative Care’ will be removed as a disposition destination and replaced by ‘Comfort Care’ with options of home palliative support or palliative care unit so as to avoid perpetuating misconceptions of palliative care and life-sustaining care as mutually exclusive. The application will also have functionality that allows users to click on terms or words that are confusing to allow for greater explanation. Our web-app will directly integrate our HF prediction model into the interface and also allow for alternative presentations of numerical risk—such as the use of simulated experiences to convey probabilistic information.^49^ Finally, it is important to make decision tools that are easy to use at bedside, and while well received, a paper-based tool would prove too cumbersome for day-to-day clinical use. After converting My-HF into a web-app we will conduct a pilot randomized trial in preparation for an anticipated definitive large multi-centre randomized controlled trial to evaluate the impact of My-HF on an array of patient reported outcome and experience measures (PROMs and PREMs).

## Conclusion

In conclusion, patients and SDMs found My-HF to be useful for communicating HF risk and individualized prognostic information. After conversion to a web-app we will assess the effect of My-HF on patient and SDM experience measures including satisfaction, regret, and readiness to engage in advanced care planning.

## Supplemental Material

sj-pdf-1-gos-10.1177_21514593211050513 – Supplemental Material for Qualitative Evaluation of a Novel Educational Tool to Communicate Individualized Hip Fracture Prognostic Information to Patients and Surrogates: My Hip Fracture (My-HF) Click here for additional data file. Supplemental Material, sj-pdf-1-gos-10.1177_21514593211050513 for Qualitative Evaluation of a Novel Educational Tool to Communicate Individualized Hip Fracture Prognostic Information to Patients and Surrogates: My Hip Fracture (My-HF) by Corita Vincent, Pete Wegier, Vincent Chien, Allison Miyoshi Kurahashi, Shiphra Ginsburg, Hedieh Molla Ghanbari, Jesse Isaac Wolfstadt and Peter Cram in Geriatric Orthopaedic Surgery & Rehabilitation
