# Supplementary material for: Qualitative Evaluation of a Novel Educational Tool to Communicate Individualized Hip Fracture Prognostic Information to Patients and Surrogates: My Hip Fracture (My-HF)
Source: Geriatr Orthop Surg Rehabil. 2021 Oct 23;12:21514593211050513. doi: 10.1177/21514593211050513 (PMC8543715; doi:10.1177/21514593211050513)
Supplement: sj-pdf-1-gos-10.1177_21514593211050513 – Supplemental Material for Qualitative Evaluation of a Novel Educational Tool to Communicate Individualized Hip Fracture Prognostic Information to Patients and Surrogates: My Hip Fracture (My-HF) [file sj-pdf-1-gos-10.1177_21514593211050513.pdf]

Supplemental File 1: My-Hip Fracture Tool

Personalized Hip Fracture Treatment Information

1 HIP JOINT ANATOMY AND FRACTURE RISK FACTORS

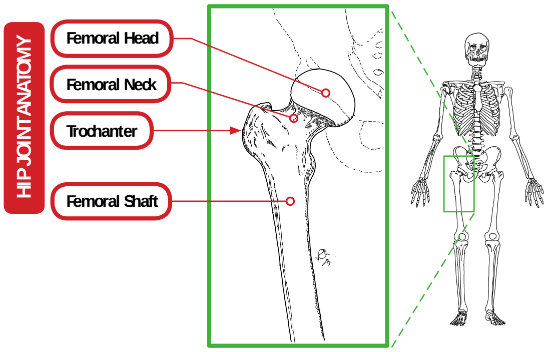

You / your family were admitted to hospital with a hip fracture.

COMMON RISK FACTORS

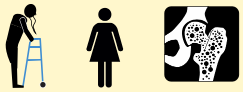

Older Age | Female Sex | Osteoporosis

It is important to remember that many hip fractures happen in patients **without any risk factors.**

2 HIP FRACTURE TREATMENTS

Your hip fracture was treated using:

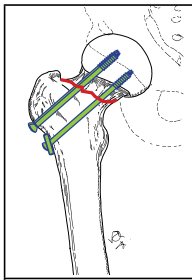

INTERNAL FIXATION Cancellous Screws ◯

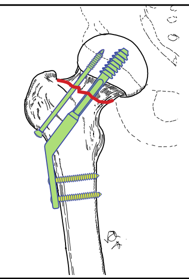

INTERNAL FIXATION Dynamic Hip Screw ◯

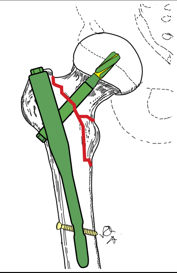

INTERNAL FIXATION Intramedullary Nail ◯

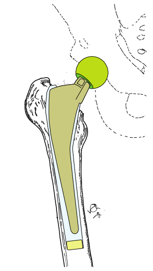

HEMI ARTHROPLASTY ◯

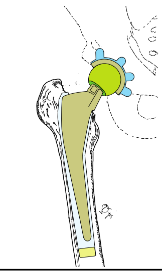

TOTAL HIP ARTHROPLASTY ◯

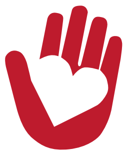

COMFORT CARE ◯

Patient Information

Affix Patient Sticker Here

Date

MM / DD / YYYY

Prepared By:

Last Name

First Name

Sinai Health System

3 YOUR INDIVIDUAL RISK

We can make some estimates about your recovery. Based on what we know ...

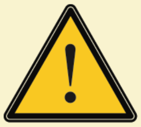

A SURGICAL COMPLICATIONS

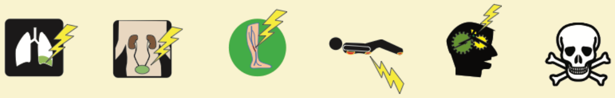

Pneumonia | Urinary Tract Infections | Blood Clots | Bedsores | Delirium | Death

Your risk of a **major complication** within 30 days of surgery is: ______ %

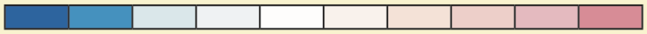

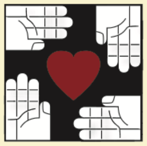

B NEED FOR REHAB OR LONG TERM CARE AFTER ACUTE CARE

Your probability of needing **Long Term Care** or **Rehabilitation** is: ______ %

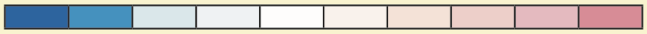

Our prediction is just a ***best guess***. Remember, *some* patients will do *much better* than this guess ... and *some* will *do worse*. Still, our guess is pretty good and should help you to understand what your recovery might be like.

4 WHAT'S NEXT?

We have decided how to treat your fracture and we have talked a bit about risk and what to expect. Based on your performance to date, it looks like once you leave hospital, you will go to ...

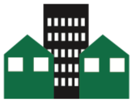

HOME

- Where you will be most comfortable
- We think your pain and recovery can be managed well here

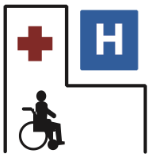

REHABILITATION

- You will get lots of physiotherapy and professional care.
- If all goes well, you could return home in a few weeks.

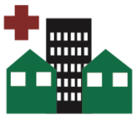

ASSISTED LIVING / LONG TERM CARE

- We recognize that you had many medical problems before your hip fracture and that most of these conditions still exist.
- In long term care, you will have the professional help you need

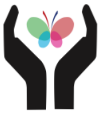

PALLIATIVE CARE

- We will focus on comfort, controlling your symptoms and maximizing quality of life.
- We acknowledge that death is likely to occur within the next few months

Supplemental File 2: Interview Guide

Interview Guide

1. Now, I am going to ask you to look at a brochure. This brochure is designed to present you with information about hip fractures. We designed the brochure because patients and families have told us previously that they don't feel like they have enough information and that the information that we give them is confusing. After patients told us this, we developed this brochure to see if it helps.
2. The brochure is organized into 4 different sections
    a. Part 1: What is a broken hip
    b. Part 2: How did my doctors fix my hip and care for me
    c. Part 3: How bad is this? (prognostic information derived from published risk calculator (http://riskcalculator.facs.org/RiskCalculator/)
    d. Part 4: What next?
3. We are going to go through each section step-by-step. As you look at each section I will ask you some questions to guide you. Specifically:
    a. What do you think of the general appearance?
        i. Does it look good? Bad? What would you change?
    b. What do you think of the writing?
        i. It the writing clear? Or confusing? Or upsetting
        ii. What would you change?
    c. What do you think of the graphics/pictures?
        i. Are they clear? Are they confusing?
        ii. What would you change?
    d. What do you think of the numbers we are presenting?
        i. Are they clear? Are they confusing?
        ii. What would you change?
        iii. How would you interpret x% risk of
    e. Overall do you find the information in this section useful
4. Overall at the end:
    a. How would you use this information
    b. Do you think it would be helpful in long term planning
    c. Is there any other information you would like to see included?

As relevant: For patients who dislike comfort care/palliative/death as possible adverse event:

- Could you tell me a bit more about what you dislike?
- ”if this was the case for you would you want to know?" why or why not

5. I will use a pen to take notes as we talk and will audio record our conversation.
6. Do you have any questions?

    a. Answer any questions that the patient/SDM has.

7. OK: Let’s begin.

    a. We will start with Part 1:

    (Proceed through Parts 1, 2, 3, and 4 of the brochure. Process will follow the order and instructions described above.

    When complete say: “OK. We are done. Do you have any other questions or comments for us? OK then. Well, thank you very much. We greatly appreciate your time and thoughts.”)

*Highlighted red text indicates questions which were added during iterative refinement of the interview guide in order to explore emerging themes.
